# Iron enhances the binding rates and translational efficiency of iron responsive elements (IREs) mRNA with initiation factor eIF4F

**DOI:** 10.1371/journal.pone.0250374

**Published:** 2021-04-21

**Authors:** Mateen A. Khan, Artem V. Domashevskiy

**Affiliations:** 1 Department of Life Science, College of Science & General Studies, Alfaisal University, Riyadh, Saudi Arabia; 2 Department of Sciences, John Jay College of Criminal Justice, The City University of New York, New York, NY, United States of America; Universidad Nacional Autonoma de Mexico, MEXICO

## Abstract

Interaction of iron responsive elements (IRE) mRNA with the translational machinery is an early step critical in the initiation of protein synthesis. To investigate the binding specificity of IRE mRNA for eIF4F, kinetic rates for the eIF4F·IRE RNA interactions were determined and correlated with the translational efficiency. The observed rate of eIF4F·FRT IRE RNA interactions was 2-fold greater as compared to eIF4F·ACO2 IRE RNA binding. Addition of iron enhanced the association rates and lowered the dissociation rates for the eIF4F binding to both IRE RNAs, with having higher preferential binding to the FRT IRE RNA. The binding rates of both eIF4F·IRE RNA complexes correlated with the enhancement of protein synthesis *in vitro*. Presence of iron and eIF4F in the depleted WGE significantly enhanced translation for both IRE RNAs. This suggests that iron promotes translation by enhancing the binding rates of the eIF4F∙IRE RNA complex. eIF4F·IRE RNA binding is temperature-dependent; raising the temperature from 5 to 25°C, enhanced the binding rates of eIF4F·FRT IRE (4-fold) and eIF4F·ACO2 IRE (5-fold). Presence of Fe^2+^ caused reduction in the activation energy for the binding of FRT IRE and ACO2 IRE to eIF4F, suggesting a more stable platform for initiating protein synthesis. In the presence of iron, lowered energy barrier has leads to the faster association rate and slower rate of dissociation for the protein-RNA complex, thus favoring efficient protein synthesis. Our results correlate well with the observed translational efficiency of IRE RNA, thereby suggesting that the presence of iron leads to a rapid, favorable, and stable complex formation that directs regulatory system to respond efficiently to cellular iron levels.

## Introduction

Iron homeostasis is critical to human health, and disruption in cellular iron regulation (either iron deficiency or excess) can lead to neurodegenerative disorders [1]. Excessive concentration of iron in the brain contributes to multiple disorders: Alzheimer’s, Parkinson’s, and Friedreich’s ataxia [2, 3]. Iron plays several physiological roles in the brain, being important for neuron myelination, neurotransmitter synthesis, mitochondrial functions and energy generation [4, 5]. Although iron regulatory mechanisms exist at the transcriptional level of gene expression, events such as iron absorption, transportation, and/or storage are rather regulated tightly at the translational level through the iron regulatory proteins (IRPs) signaling pathway [6, 7]. IRPs control iron homeostasis through their binding to the iron responsive elements (IREs). IREs are 30-nucleotide stem-loop structures that includes: terminal pseudo-tri-loop (CAGUGX), a five base pair upper helix, a variable lower helix, and a mid-stem cytosine bulge (C8) [8, 9]. IREs may be situated in either the 5’- or the 3’-untranslated regions (UTRs) of the target mRNAs [8, 10]. The IRE/IRP interactions exert control over both either mRNA translation and degradation, depending on the position of the IREs. IRP proteins bind with different strength to IREs of the IRE RNA family [11], creating a graded set of mRNA responses to iron fluctuations *in vivo*. Deletion of the 30-nt IRE leads to the removal of the IRP modulation, and represses IRP independent rate of protein synthesis [11]. In iron deficient cells, binding of the IRP to the IRE (located within the 5’-UTR of target mRNA) blocks mRNA translation by preventing the mRNA-ribosome complex from forming [6, 12]. Whereas, in cells with excessive iron levels, iron binds to both IRPs and IREs, and induces conformational changes that promote IRPs dissociation from the target mRNA. This leads to stabilization of the message, and enhancement of the protein synthesis [6]. Elevated cellular iron levels can interrupt IRP/IRE binding, and promote translation of the ferritin and ferroportin in a way that destabilizes divalent metal transporter and transferrin receptor mRNA [6]. Therefore, disturbance of the IRP/IRE signaling pathway caused by the elevated iron levels impairs iron homeostasis, thereby contributing to the protein aggregation and neuronal loss observed in Alzheimer’s and Parkinson’s diseases [13, 14]. The implicated proteins misfold, causing fibrils to form that propagate across neurons in the brain. The expression level of the amyloid precursor protein (APP) (in Alzheimer’s disease) (or α-synuclein in Parkinson’s disease) is a vital determinant of the rate of its fibrillization and neurotoxicity [14, 15]. Targeting the messenger RNA that encodes for the APP and/or α-synuclein, and selectively inhibiting mRNA translation is a potentially promising way to show the progression of Alzheimer’s and Parkinson’s disease [16]. Our earlier report [11] revealed a novel mechanism for the iron-induced regulation of mRNA translation. Iron increases the binding affinity of eukaryotic translation initiation factor eIF4F to the 5’-UTR of IRE mRNA, important for protein expression. It was suggested that binding of iron to IRE RNA induces structural changes within the RNA, thus downregulating the IRP-RNA binding. This results in the facilitated eIF4F binding, leading to a stable translation initiation complex [11].

In eukaryotes, initiation of protein synthesis begins with binding of the 5’-cap moiety of mRNA to eIF4E, the small subunit of eIF4F [17, 18]. eIF4G, the larger subunit of eIF4F, recruits additional initiation factors (eg., eIF4B, eIF4A and eIF3) that aid in the unwinding of the secondary structure within the 5’-leader sequence, thus allowing 40S ribosomal scanning; poly-(A)-binding protein stabilizes the eIF4F complex; and eIF3 promotes 40S ribosomal subunit binding and mRNA translation [19, 20]. Association of eIF4F, eIF4A and eIF4B are believed to catalyze the efficient unwinding of secondary structure in the 5’-UTR of mRNA [21]. These initiation factors promote the functional circularization of mRNA necessary for efficient translation. In eukaryotic cells, non-coding mRNA structures make major contributions to the translation initiation rates and gene expression levels. For example, the ability of IRE RNA to bind both the specific protein synthesis inhibitor (IRP) and a generic protein synthesis enhancer (eIF4F) [11, 22]. We showed that metal ions decrease the stability of IRP/IRE, and simultaneously, increase the eIF4F/IRE binding [11, 22]. The ability to form an activator (eIF4F/IRE) complex, or a repressor (IRE/IRP) complex will be influenced by the kinetics of the protein binding, and how this binding is affected by iron levels. Conformational changes in IRP structure play an important role in IRE RNA binding [23]. Two IRE RNA molecules bind a single IRP via comparable RNA-protein surface area contacts. Their stem loop conformations are different however [24], suggesting different binding mechanism influenced by the conformational plasticity of RNA and protein.

Earlier studies [11, 22] have shown that IRE RNA interact with both translation initiation factor eIF4F and IRP, forming a stable complex. IRP and eIF4F compete for IRE RNA binding. These observations suggest that interactions of IRE RNA with eIF4F is an important step in iron regulation of protein synthesis. Here we report the temperature-dependent kinetic parameters for the binding of eIF4F to FRT IRE and ACO2 IRE, and correlate these binding rates with translational efficiency of the mRNA. We demonstrate that iron enhances kinetic rates of two IREs with eIF4F, on the other hand, iron lowers the two IRE RNA molecules binding rates to IRP [25]. Moreover, we observed a recovery of the ferritin and mitochondrial aconitase IRE mRNA protein synthesis after adding exogenous eIF4F into the eIF4F-depleted cell extracts of wheat germ. Iron increases the rate of association and decreases the rate of dissociation for IRE RNA binding to eIF4F. Addition of iron significantly reduces the activation energy barrier of eIF4F∙IRE RNA complex formation.

## Materials and methods

### Preparation of RNA and protein

**Fig 1:**
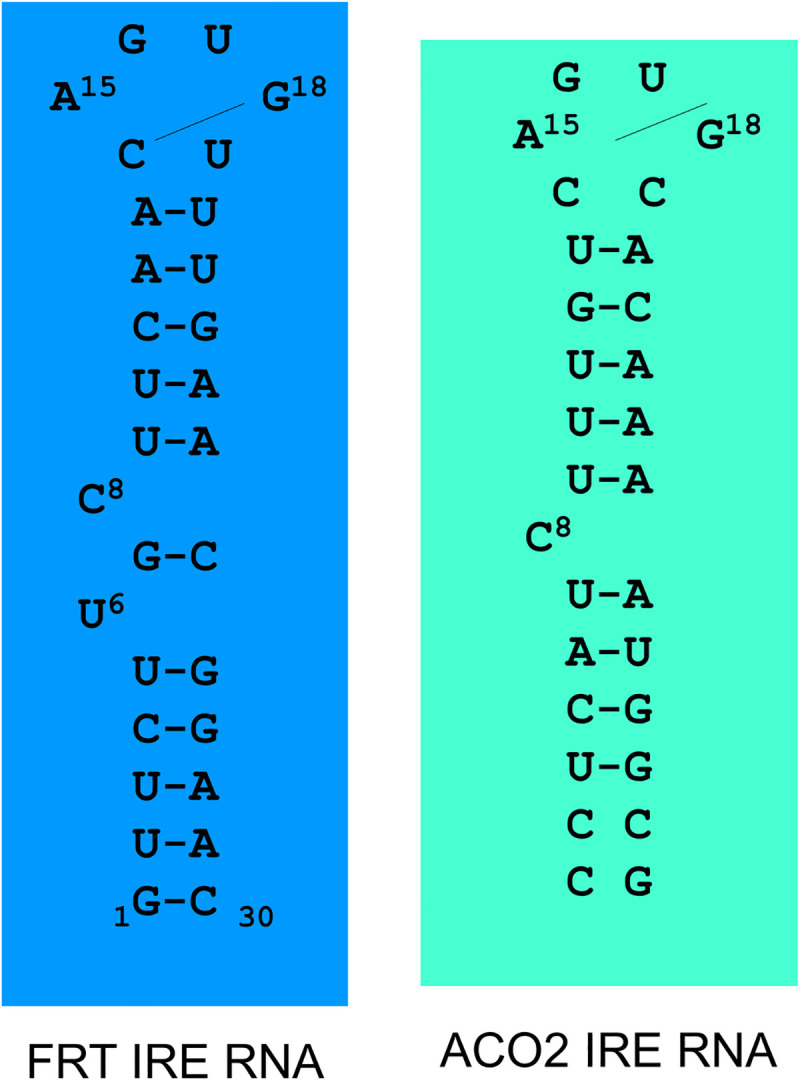
Secondary structures of ferritin and mitochondrial aconitase IREs.

Ferritin and mitochondrial aconitase IRE RNA (30-nt) were acquired from Metabion International AG, (Germany). For kinetic studies, ferritin IRE RNA (sequence: -GUU CUUGCUUCAACAGUGUUUGAACGGAAC) and mitochondrial aconitase IRE RNA (sequence: -CCUCAUCUUUGUCAGUGCACAAAAUGGCG) were melted and reannealed as described previously [22, 26]. Secondary structures of ferritin and mitochondrial aconitase IREs are shown in Fig 1 [26, 27]. Capped ferritin and mitochondrial aconitase mRNAs were transcribed according to the method described previously [28–30]. Reaction conditions and purification of synthesized RNA is described in the protocol for the Promega. The purity of synthesized RNA was confirmed by measuring the absorbance ratio A_260_/A_280nm_ in diethylpyrocarbonate (DEPC)-treated water. Wheat germ (WG) extract was purchased from Promega, (WI, USA). RNA was quantified spectrophotometrically by measuring absorbance at 260 nm (having standard of 40 μg/ml RNA as 1). The eIF4F protein was purified from rabbit reticulocyte lysate, and quantified as described previously [31]. Protein concentration was measured by Bradford method [32]. The pT7-luciferase reporter construct, in which the firefly *luc*-coding region is under the control of the T7 promoter in a pGEM vector (Promega), have been described previously [33]. The IRE containing region were introduced into the *Hind* III and *Bgl* II sites within the polylinker and the *luc* reporter introduced into the *Sal* I and *Sma* I sites. DNA was linearized with *Sal* I, a site immediately upstream of the *luc* open reading frame. IRE luciferase reporter, a 1739 bp fragment containing the gene coding for luciferase from a pGEM-*luc* vector (Promega) was amplified by PCR and blunt end ligated into a vector containing the IRE sequence as described previously [33]. The resulting plasmid containing the IRE eighteen bases upstream of the luciferase start codons, and RNA was synthesized using Promega RiboMax^TM^ large scale RNA production system T7 following the manufacturer’s protocol. The capped RNA was synthesized using the m^7^GpppG cap analogue under conditions optimized to ensure that more than 95% of the RNA was capped [28, 29, 34].

### Stopped-flow kinetic measurements

Stopped-flow spectrophotometer (instruments dead time was 1-millisecond) was used to measure the kinetics of FRT and ACO2 IRE interaction with eIF4F with and without addition of iron. Fluorescence intensity (measured in Volts) of eIF4F and eIF4F∙IRE RNA complex in titration buffer (20 mM HEPES/KOH, pH 7.6 containing 150 mM KCl) were measured (λ_ex_ = 280 nm and λ_em_ = 334 nm). In each sample, instrument collected about 1000 data points. Time-dependent fluorescence signal was measured up to 250-milli-second. Prior to the data collection, all samples were incubated for 15 minutes, allowing for equilibration to the experimental temperature using temperature-controlled circulating water bath. After rapid mixing of eIF4F protein with IRE RNA, the time course of the intrinsic fluorescence intensity (in V) was recorded by computer data acquisition. The intrinsic fluorescence intensity of eIF4F decreased when bound to IRE RNA. Binding rates of eIF4F·IRE RNA interactions were measured at saturating concentration of RNA. The concentration of eIF4F in first syringe was 0.2 μM (0.1 μM after mixing). The concentrations of IRE RNA and Fe^2+^ in the second syringe were 2.0 μM and 100 μM, respectively (1.0 μM and 50 μM after mixing, respectively). In control experiments, rapid mixing of 0.1 μM (final) eIF4F with 50 μM (final) Fe^2+^ alone did not change the fluorescence signal. Samples were passed through 0.22-μm filter and degassed prior to loading into syringes. Kinetic traces are the average of three sets of experiment. KaleidaGraph (Synergy software, version 2.1.3) was used to fit the average set of data.

### Kinetic data analysis and curve fitting

Stopped-flow fluorescence data, representing eIF4F binding to IRE RNA were analyzed using curve-fitting program, Global analysis software as described previously [34]. Stopped-flow data points were plotted according to a single- and double-exponential fit. The observed rate constant for the single exponential fit were derived from the following relations [35–37]:

$$F_{t}=R\times e^{\left(-k_{obs}\times t\right)}+F_{f}$$ (Eq 1)

where, *F*_*t*_ is the fluorescence over time t and *F*_*f*_ is the final change in fluorescence. *k*_obs_ is the observed rate. The observed rate constants for the double-exponential fits were derived from the following relations:

$$F_{t}=R_{1}\times e^{\left(-k_{obs_{1}}\times t\right)}+R_{2}\times e^{\left(-k_{obs_{2}}\times t\right)}+F_{f}$$ (Eq 2)

where *R*_1_ and *R*_2_ are the amplitudes of the two exponentials, and *k*_obs1_ and *k*_obs2_ are the observed rate constants for the first and second components of the double exponential fits. The subscripts 1 and 2 refer to the fast and slow phases, respectively. Stopped flow data were fit to a single and double exponential function using Kaleida Graph software as described previously [36–38].

To assess the activation energy of the protein-RNA complexes with and without addition of iron, the temperature-dependent observed rate constants (*k*_obs_) data was analyzed according to the following relations:

$$\ln k_{obs}=\left(\frac{E_{a}}{RT}\right)+\ln A$$ (Eq 3)

where *k* is the observed rate constant, *E*_a_ is the activation energy, *R* [8.314 J/K. mol] is the universal gas constant, T (Kelvin) is the absolute temperature, and *A* is the Arrhenius pre-exponential factor. The value of *E*_a_ was calculated from the slope of the fitted linear plot of ln*k versus* T^-1^.

### Dissociation rate constants measurements

Dissociation rate constant of the eIF4F·IRE RNA complexes was measured by spectrofluorometer with the steady state condition as described previously [34, 39, 40]. Protein sample was excited at 280 nm (4-nm slit width), and the fluorescence emission was monitored at 340 nm (5-nm slit width). IRE RNA and eIF4F protein samples were incubated at 20°C in titration buffer for about 15 minutes to ensure complex equilibrium. Concentrations of eIF4F, IRE RNA, and Fe^2+^ in a reaction sample were 0.1 μM, 1 μM, and 50 μM, respectively. The dissociation rate constant for IRE RNA was measured by rapid mixing of eIF4F·IRE RNA complex with 15-fold excess of 20 mM HEPES/KOH (pH 7.6) buffer in the cell; dissociation was monitored as the fluorescence signal increase. The increasing signal in the dissociation portion of fluorescence data was fit to the single-exponential function using Kaleida Graph software (version 2.1.3) as described previously [38, 40].

### *In vitro* translation assay

eIF4F was assayed for its ability to stimulate translation in the wheat germ assay using eIF4F-depleted cell extract of wheat germ. For all *in vitro* translation assays, the transcript capped ferritin and mitochondrial aconitase IRE luciferase reporter RNA was heated to 65°C for 5 minutes and cooled slowly to 25°C for 30 minutes for the sample to annealed completely. When iron was used, anaerobic conditions for Fe^2+^ were obtained with sealed glass vials purged with nitrogen. Wheat germ (WG) extract mixtures were prepared according to the manufacturer’s instructions. Depleted wheat germ (Dep WG) extracts of eIF4F/eIFiso4F were prepared as described previously [38, 41, 42]. eIF4F/eIFiso4F depleted cell extract of WG was generated by continuous mixing of 200 μl WG extract with 300 μl of m^7^-GTP-Sepharose at 4°C for 30 min. The lysate was separated by a spin column. The column resin was then washed with 1000 μl of 20 mM HEPES/KOH, pH 7.6 buffer, followed by resuspension of the resin in the buffer that contains 100 mM GTP. GTP-eluted fractions were collected, and the depletion of wheat germ extract was confirmed by the SDS-PAGE and the Coomassie Blue staining as shown previously [34]. Briefly, 200 μl reaction sample included 1 μg of IRE-luc RNA (capped FRT or ACO2) template, 10 μM amino acids and 50 units RNase inhibitor with WG extract or depleted WG extract, and incubated at 25°C for 30 min as described previously [40, 41]. To investigate the eIF4F-dependent translation, depleted cell extracts of WG were supplemented with eIF4F protein. eIF4F and Fe^2+^ concentrations were 10 nM and 50 μM, respectively. Translation reaction mixtures were incubated for 2 h at 25°C, after which protein production was assayed. Translation of ferritin and mitochondrial aconitase was quantified by measuring the absorbance at 495 nm, after addition of 500 μM luciferin. Brome mosaic virus RNA provided by Promega was used as a control. For all protein synthesis assay measurements, each sample was repeated at least three times, and the average value is reported.

## Results

**Fig 2:**
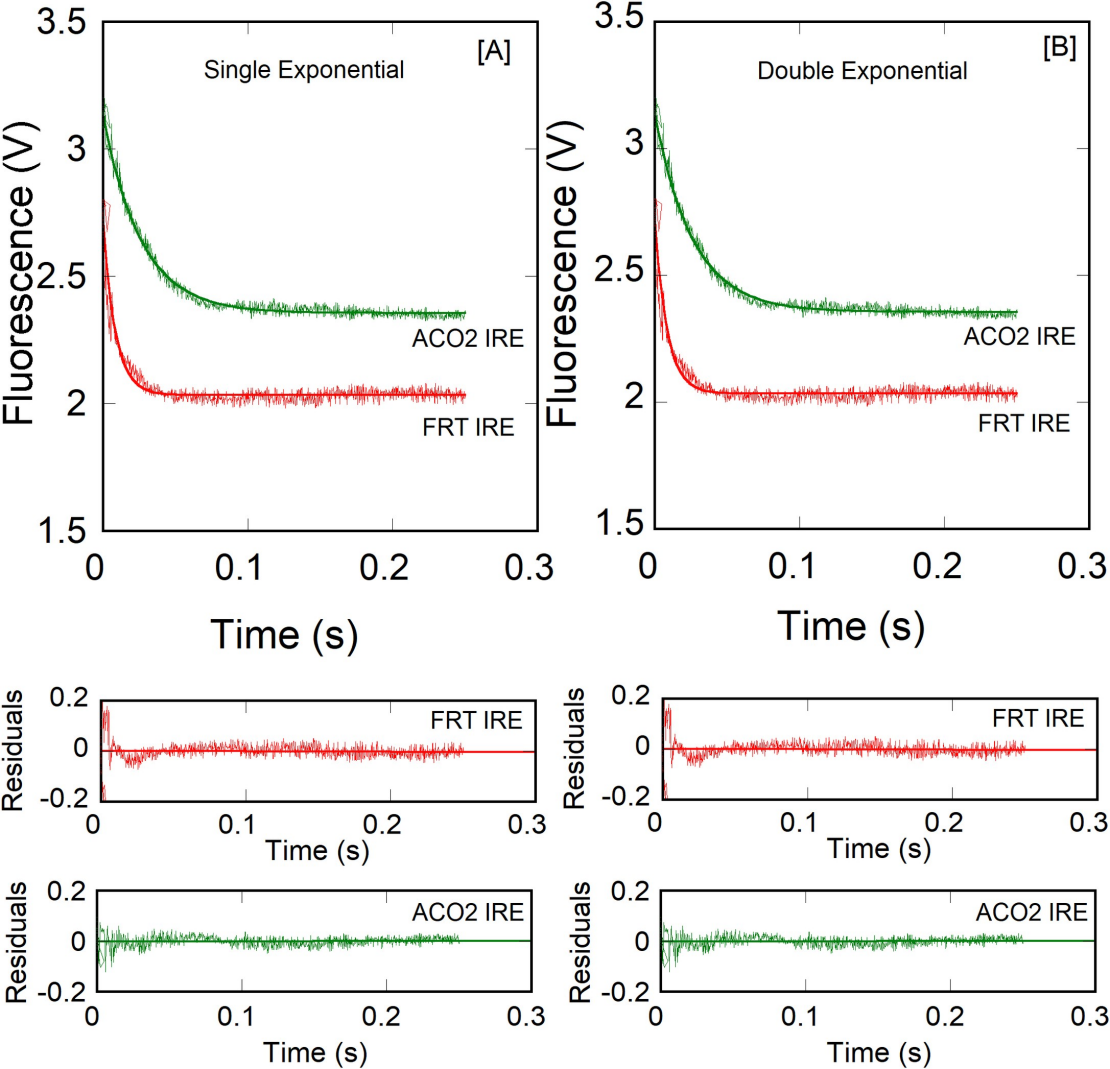
Kinetic analyses of IRE RNA interactions with eIF4F. A solution of 1.0 μM (after mixing) FRT (or ACO2 IRE RNA) was rapidly mixed with 0.1 μM (after mixing) eIF4F at 20 ˚C. (A) Single-exponential fit to the kinetic data. (B) Double-exponential fit to the same kinetic data as in panel (A). A double exponential fit did not improve the fitting. Solid lines represent the fitted curve drawn through the data points and were best fit by assuming a single-exponential fit. Fluorescence is measured in volts (V). Residuals for the corresponding fits are shown in the *lower panels*. The observed rate constant for FRT and ACO2 IRE RNA binding to eIF4F were significantly different.

**Fig 3:**
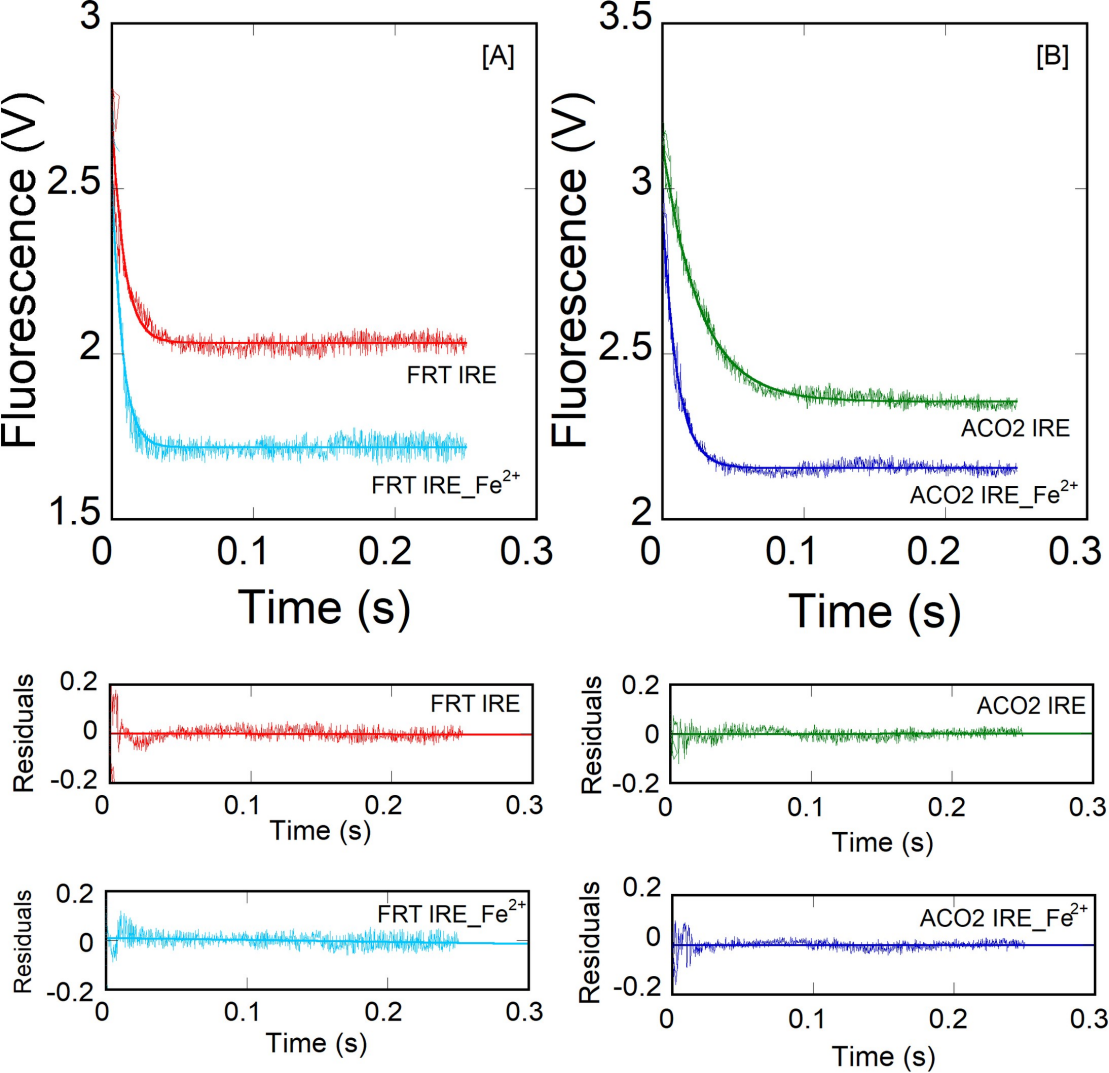
Iron enhances kinetic rates for the interaction of IRE RNA with eIF4F. eIF4F at 0.1 μm (final) was mixed with (A) FRT IRE RNA (B) ACO2 IRE RNA with or without addition of 50 μM (final) Fe^2+^ at 20 ˚C. Concentration of IRE RNA was 1 μM (final). Solid line represents the fitted line drawn through the data points was fit by assuming a single exponential process. Residuals for the fits are shown in the *lower panels*. Addition of iron increases the binding rates of FRT and ACO2 IRE RNA-eIF4F complex 2-fold.

Interactions between ferritin IRE RNA and initiation factor eIF4F have been reported previously [11]. To further gain insight into eIF4F·IRE RNA specificity, the effect of temperature on the kinetic measurements of eIF4F binding to ACO2 IRE RNA were examined and compared to the FRT IRE RNA with and without addition of iron. Fig 2 shows the stopped-flow traces for the rapid mixing of 1.0 μM FRT or ACO2 IRE RNA with 0.1 μM eIF4F (final concentrations). Kinetics of IRE RNA binding to eIF4F were measured under pseudo-first order conditions, where IRE RNA was in excess and eIF4F was limiting. The stopped-flow fluorescence data (Fig 2) show that the rate constant of eIF4F binding to FRT IRE RNA was ~2-fold faster than ACO2 IRE RNA (eIF4F**∙**FRT IRE, *k*_obs_ = 106 ± 6.8 s^-1^; eIF4F**∙**ACO2 IRE, *k*_obs_ = 49 ± 2.6 s^-1^). We have observed previously [11] that metal ions stabilized the eIF4F**∙**FRT IRE RNA complex, while decreasing the stability of IRE∙IRP complex. Here we show the effects of iron on binding rates of FRT and ACO2 IRE RNA with eIF4F (Fig 3). Adding iron, increased (2-fold) the observed rate constant (*k*_*obs*_) for the binding of eIF4F with FRT and ACO2 IRE (eIF4F**∙**FRT IRE RNA-Fe^2+^, *k*_obs_ = 267 ± 8.8 s^-1^; eIF4F**∙**ACO2 IRE RNA-Fe^2+^, *k*_obs_ = 96 ± 6.7 s^-1^; Table 1) at 20 ˚C. Kinetic traces followed single-exponential fits over the time course measurements. The residuals (Figs 2 and 3, lower panel) did not vary over the time course, nor they diminished by a double-exponential fit (Fig 2, lower panel).

**Table 1:** Kinetic constants for the binding of iron responsive elements (IREs) mRNA with eIF4F.

Complex	Observed Rate Constant, *k*_obs_ (s^-1^)					*E*a (kJ/mol)
	5°C	10°C	15°C	20°C	25°C	
FRT IRE-RNA/eIF4F	37 ± 1.7	57 ± 3.5	81 ± 4.3	106 ± 6.8	151 ± 7.8	52.7 ± 3.4
FRT IRE-RNA-Fe^2+^/eIF4F	88 ± 2.9	119 ± 4.5	151 ± 6.4	267 ± 8.8	305 ± 9.7	38.1 ± 2.7
ACO2 IRE-RNA/eIF4F	13 ± 1.1	24 ± 1.4	35 ± 2.7	49 ± 2.6	71 ± 4.3	56.8± 2.8
ACO2 IRE-RNA-Fe^2+^/eIF4F	35 ± 1.4	52 ± 2.7	77 ± 4.4	96 ± 6.7	127 ± 8.9	42.9 ± 3.3

Temperature-dependent observed rate constants obtained from rapid mixing of iron responsive elements (IREs) mRNA with eIF4F in the absence and presence of 50 μM Fe^2+^.

**Fig 4:**
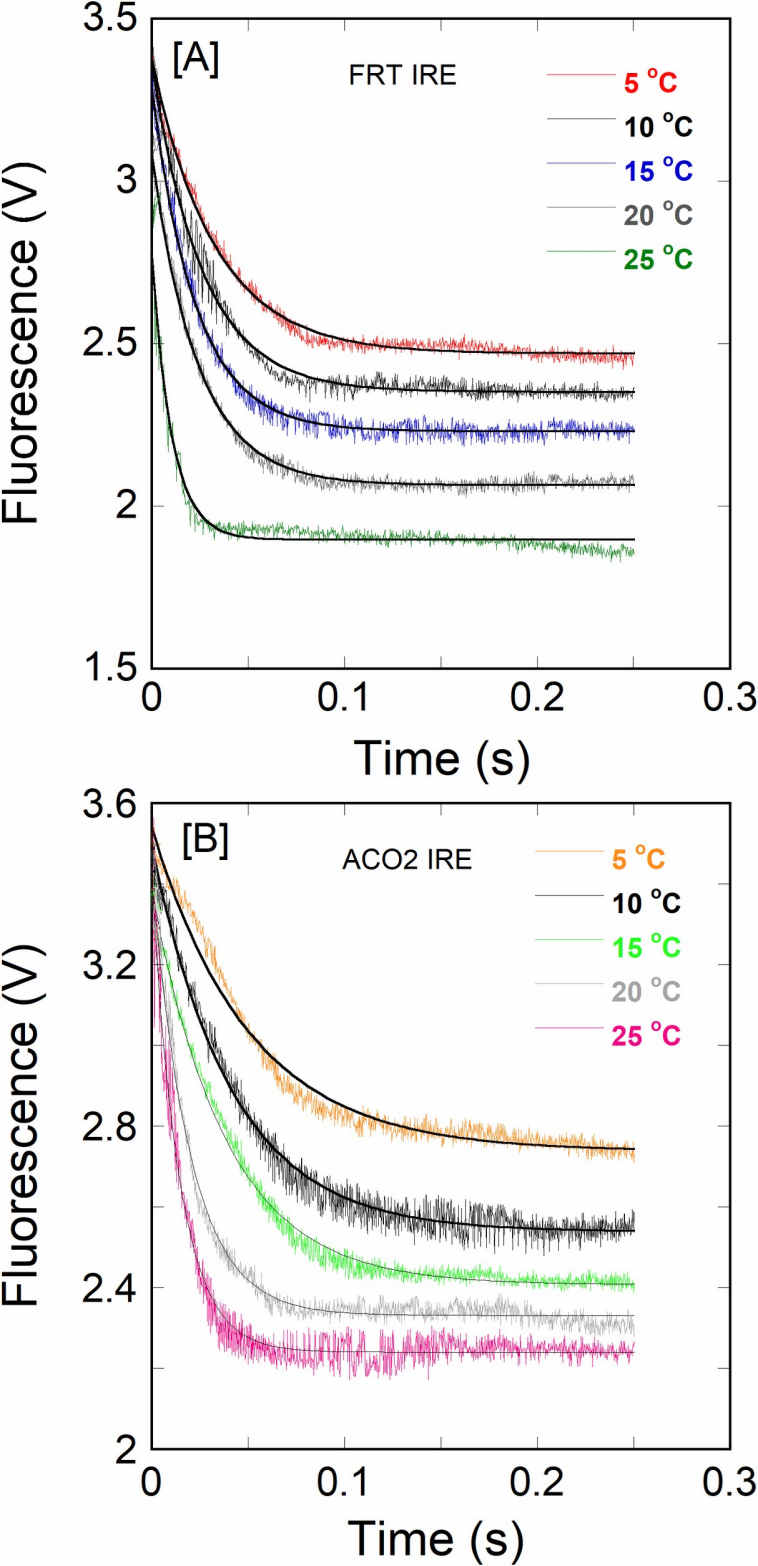
Temperature-dependent kinetic measurements of IRE RNA binding to eIF4F. Kinetic plots show the time course for the interaction of eIF4F at 0.1 μM (final) mixed with 1.0 μM (final) of (A) FRT IRE RNA and (B) ACO2 IRE RNA, and the complex formation was measured by stopped-flow. The curve (solid line) represents the fitted line drawn through the data points that was fit by assuming a single exponential process. For the FRT IRE at 5, 10, 15, 20, and 25°C, *k*_obs_ was 37, 57, 81, 106, and 151 s^-1^, respectively; for the ACO2 IRE was 13, 24, 35, 49, and 71 s^-1^, respectively. The binding rates of FRT and ACO2 IRE RNA with eIF4F increase as temperature elevates.

**Fig 5:**
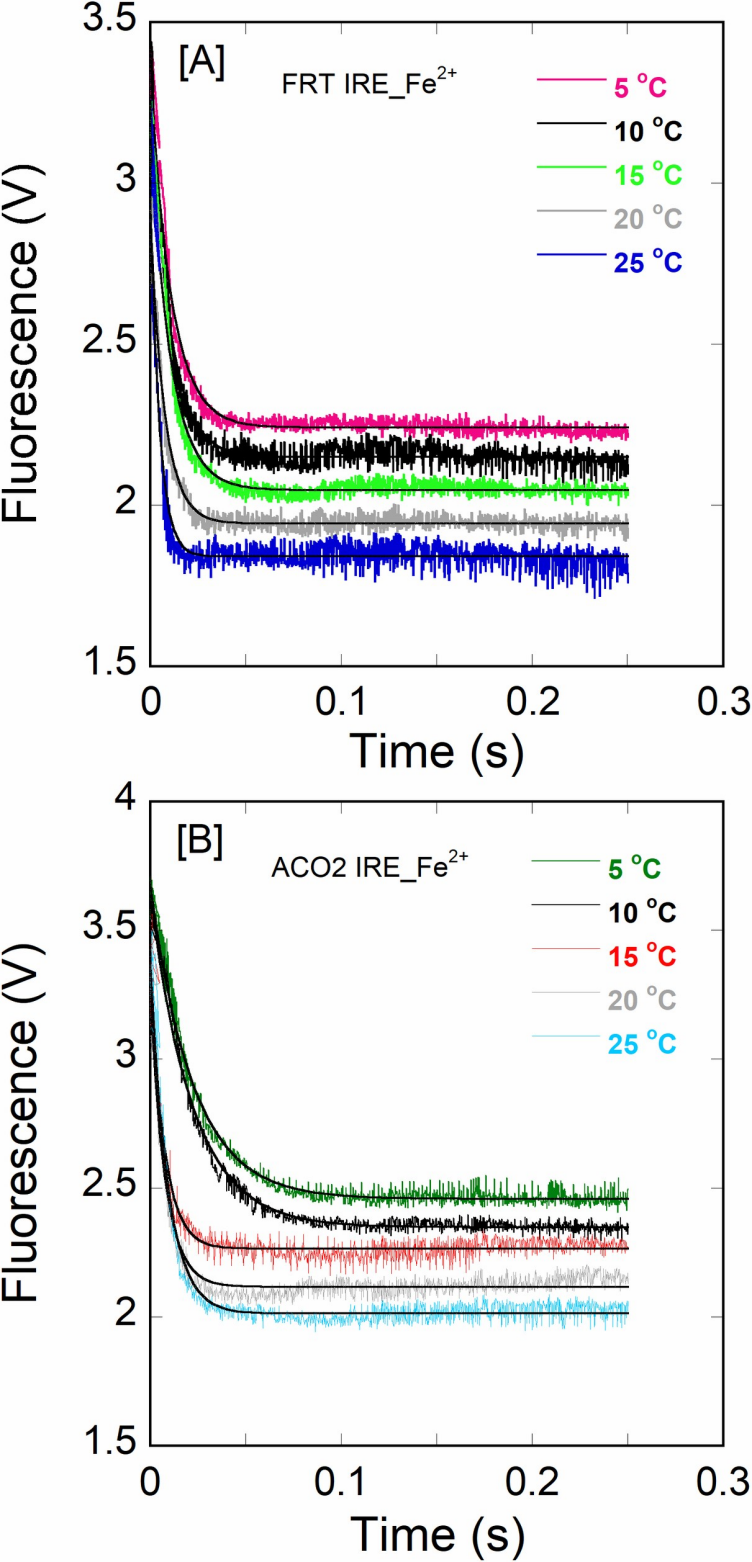
Temperature-dependent kinetic measurements of IRE RNA binding to eIF4F in the presence of iron. Kinetic plots show the time course of the interaction of eIF4F at 0.1 μM (final) mixed with (A) FRT IRE RNA-Fe^2+^ and (B) ACO2 IRE RNA-Fe^2+^. Final concentration of FRT or ACO2 IRE RNA was 1.0 μM and Fe^2+^ concentration was 50 μM. The curve (solid line) represents the fitted line drawn through the data points was fit by assuming a single exponential process. The binding rates of FRT and ACO2 IRE RNA-eIF4F complex with addition of iron at 25°C were significantly faster as compared to the rates at 5°C.

Furthermore, the temperature-dependent kinetic analysis of both IRE RNAs with eIF4F in the presence and absence of iron was conducted to determine the binding rates for the eIF4F·IRE RNA interactions. Figs 4 and 5 show the effect of temperature on the kinetics of FRT and ACO2 IRE RNA binding to eIF4F. Kinetic traces followed a single exponential fit over the range of experimental time. The observed binding rates (*k*_obs_) for the interactions of FRT or ACO2 IRE RNA with eIF4F increased as temperature elevated. The values of *k*_obs_ for the FRT IRE and ACO2 IRE RNA binding to eIF4F enhanced from 37 ± 1.7 s^-1^ to 151 ± 7.8 s^-1^, and from 13 ± 1.1 s^-1^ to 71 ± 4.3 s^-1^ (from 5°C to 25°C) (Table 1). Analysis of the kinetic data shows that the binding rates of the eIF4F∙FRT IRE RNA complex and eIF4F·ACO2 IRE RNA complex at 25°C were significantly faster than at 5°C (Table 1). Fig 5 shows the effect of temperature on the kinetic of FRT and ACO2 IRE RNA binding to eIF4F with addition of iron. The kinetic rate for the eIF4F·FRT IRE RNA-Fe^2+^ complex increased from 88 ± 2.9 s^-1^ to 305 ± 9.7 s^-1^, while the kinetic rate of eIF4F·ACO2 IRE RNA-Fe^2+^ complex increased from 35 ± 1.4 s^-1^ to 127 ± 8.9 s^-1^ (from 5°C to 25°C) (Table 1). Analyses showed that the binding rates of the eIF4F·IRE RNA-Fe^2+^ complex at 25°C were about 3.5-fold faster as compared to 5°C (Table 1). The temperature-dependent kinetic data showed that ferritin IRE RNA had consistently higher binding rates compared with the mitochondrial aconitase IRE RNA with and without iron.

**Fig 6:**
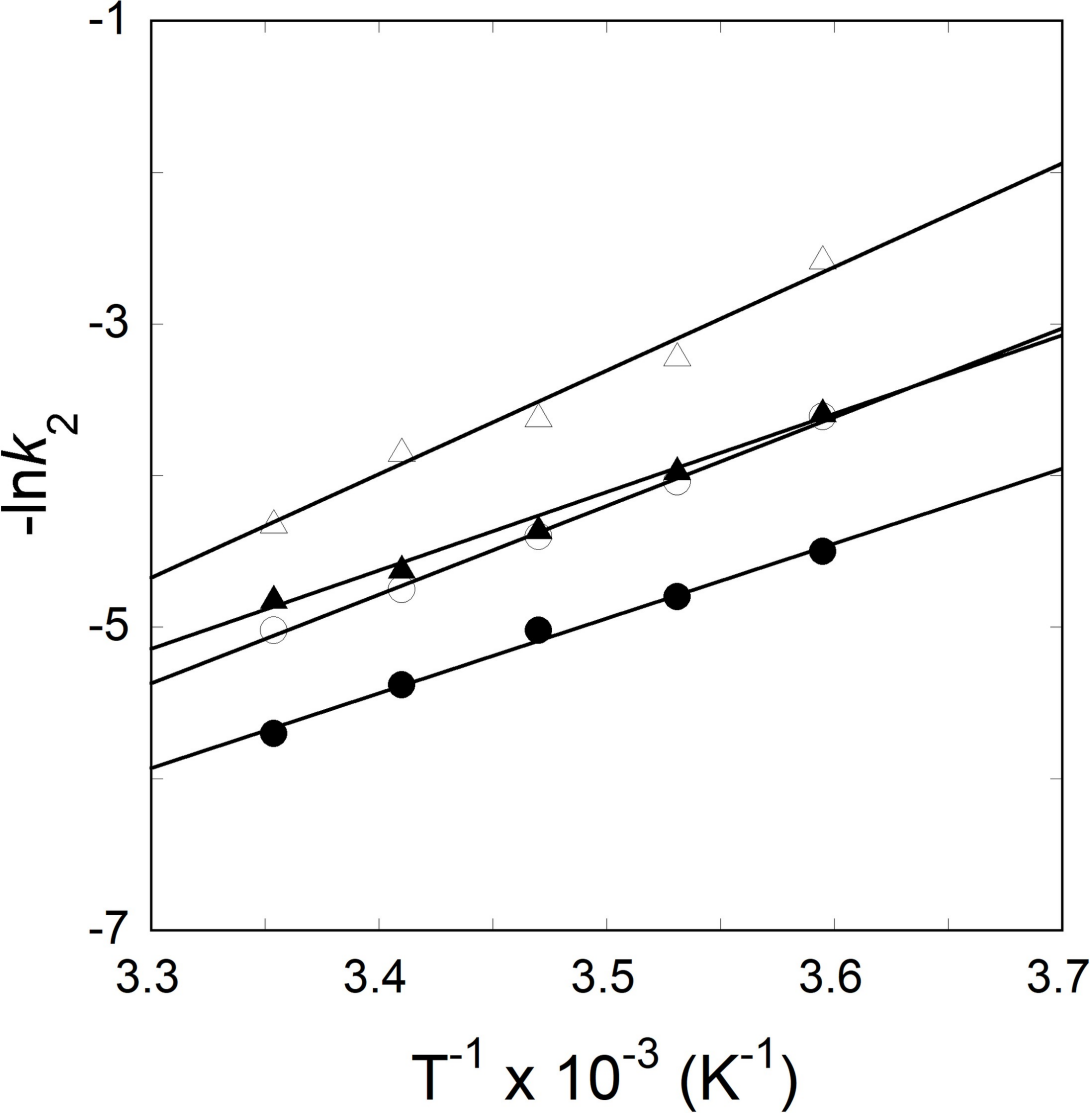
Arrhenius plots to determine the activation energies of the eIF4F∙FRT IRE RNA and eIF4F∙ACO2 IRE RNA interactions in the absence and presence of Fe^2+^. The observed rate constants for the interaction of eIF4F with FRT IRE RNA (—О—), FRT IRE RNA-Fe^2+^ (—●—), ACO2 IRE RNA (—Δ—), and ACO2 IRE RNA-Fe^2+^ (—▲—). Concentrations of eIF4F, IRE RNA and Fe^2+^ were 0.1 μM, 1 μM and 50 μM, respectively, after reactants were mixed. The activation energy was calculated from the slope of the fitted linear plot of ln *k* versus 1/*T* (Kelvin).

The observed rate constant (*k*_obs_) values of IRE RNA binding to eIF4F were determined from the kinetic data over a temperature range of 5 to 25°C. The activation energies (*E*_a_) of the IRE RNA binding to eIF4F was determined by plotting the observed rate (*k*_obs_) *versus* temperature (Fig 6). Fitting temperature dependent kinetic data to the plot of Arrhenius equation yielded the activation energy values of 52.7 ± 3.4 kJmol^-1^ and 56.8 ± 2.8 kJmol^-1^, respectively, for FRT IRE and eIF4F∙ACO2 IRE RNA complexes. Addition of iron significantly changes *E*_a_ of binding for the eIF4F·FRT IRE to 38.1 ± 2.7 kJ.mol^-1^ and eIF4F∙ACO2 IRE RNA to 42.9 ± 3.3 kJ.mol^-1^ (Table 1). Reduction in *E*_a_ of binding for the eIF4F·FRT IRE and eIF4F∙ACO2 IRE complexes with addition of iron, suggests that iron induces conformational changes in both eIF4F·IRE RNA complexes, allowing translation initiation factor eIF4F to bind, subsequently upregulating protein synthesis.

**Fig 7:**
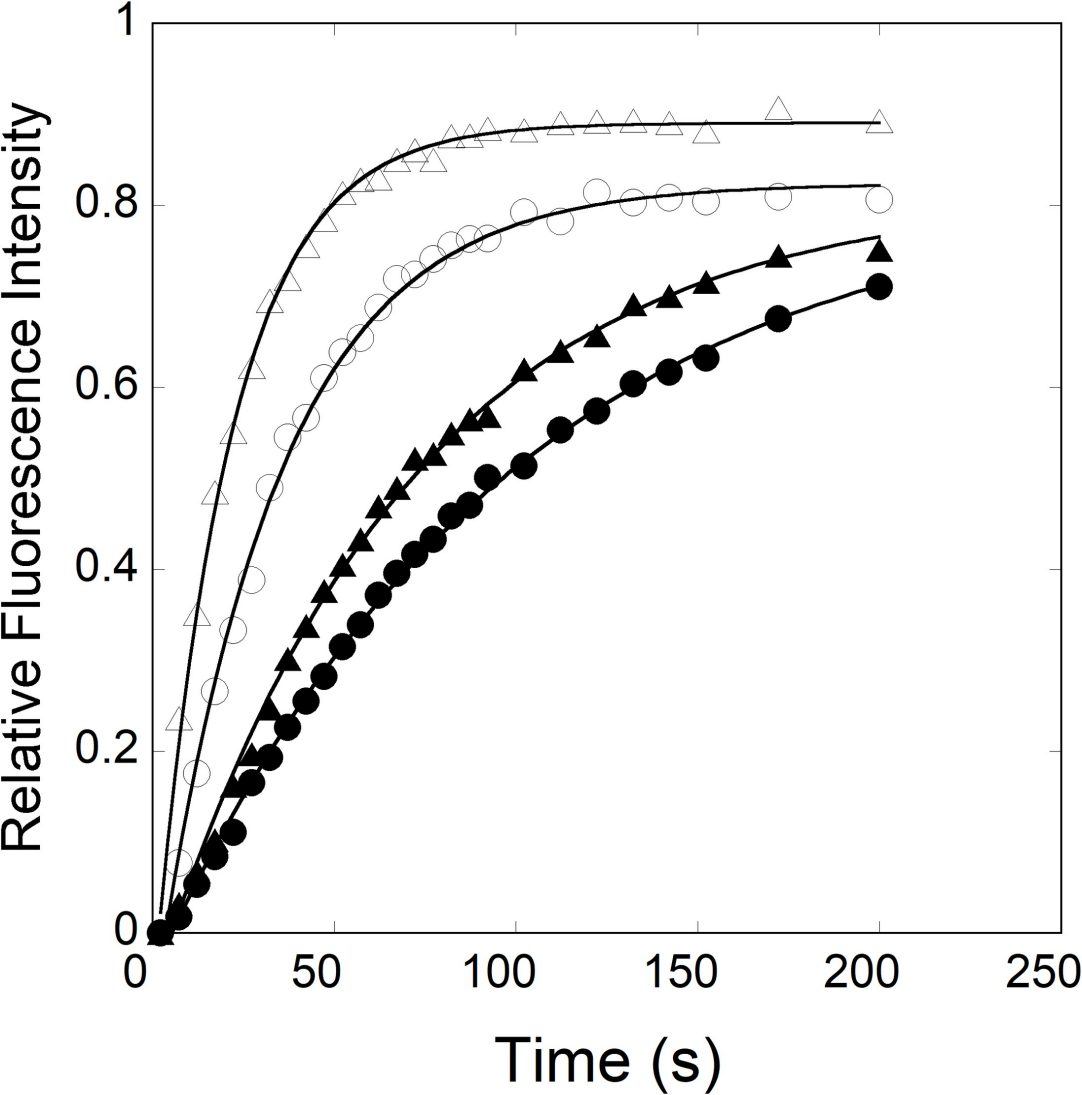
Kinetics of the IRE RNA dissociation from the pre-formed eIF4F∙IRE RNA complexes in the absence and presence of iron. Dissociation rate constant (*k*_-2_) for eIF4F∙FRT IRE RNA (—О—), eIF4F∙FRT IRE RNA-Fe^2+^ (—●—), eIF4F∙ACO2 IRE RNA (—Δ—), and eIF4F∙ACO2 IRE RNA-Fe^2+^ (—▲—) complexes were monitored by rapidly diluting 100 μl of the complex with 1500 μl of buffer at 20 ˚C. Concentrations of IRE RNA, eIF4F and Fe^2+^ were 1μM, 0.1 μM and 50 μM, respectively, after reactants were mixed.

To further understand the kinetic mechanism of this reaction, the effect of iron on the dissociation rates (*k*_-2_) of the eIF4F·FRT IRE and eIF4F∙ACO2 IRE complexes were investigated. Dissociation (*k*_-2_) of the pre-formed eIF4F∙IRE RNA complex obtained by the 15-fold diluting with buffer in the fluorescence cuvette. Fig 7 shows the dissociation curve for both eIF4F·IRE RNA complexes in the absence and presence of iron. The kinetic traces followed single-exponential fits, dissociation kinetics determined an increase in the fluorescence upon complex dissociation. The dissociation rate constants for eIF4F·FRT IRE and eIF4F∙ACO2 IRE complexes were obtained from the fitted curves as (38.0 ± 1.7) x 10^−3^ s^-1^ and (62.7 ± 4.3) x 10^−3^ s^-1^. Addition of Fe^2+^ decreased 4- and 3-fold dissociation rates for eIF4F·FRT IRE RNA [(*k*_-2_ = 10.0 ± 0.5) x 10^−3^ s^-1^] and eIF4F∙ACO2 IRE RNA [(23.0 ± 0.8) x 10^−3^ s^-1^] complexes. These results indicate that iron can selectively decrease the dissociation rates of both IRE RNA∙eIF4F complexes and increase binding rates. This suggests that eIF4F·IRE RNA complex forms more rapidly and dissociates slower in the presence of iron.

**Fig 8:**
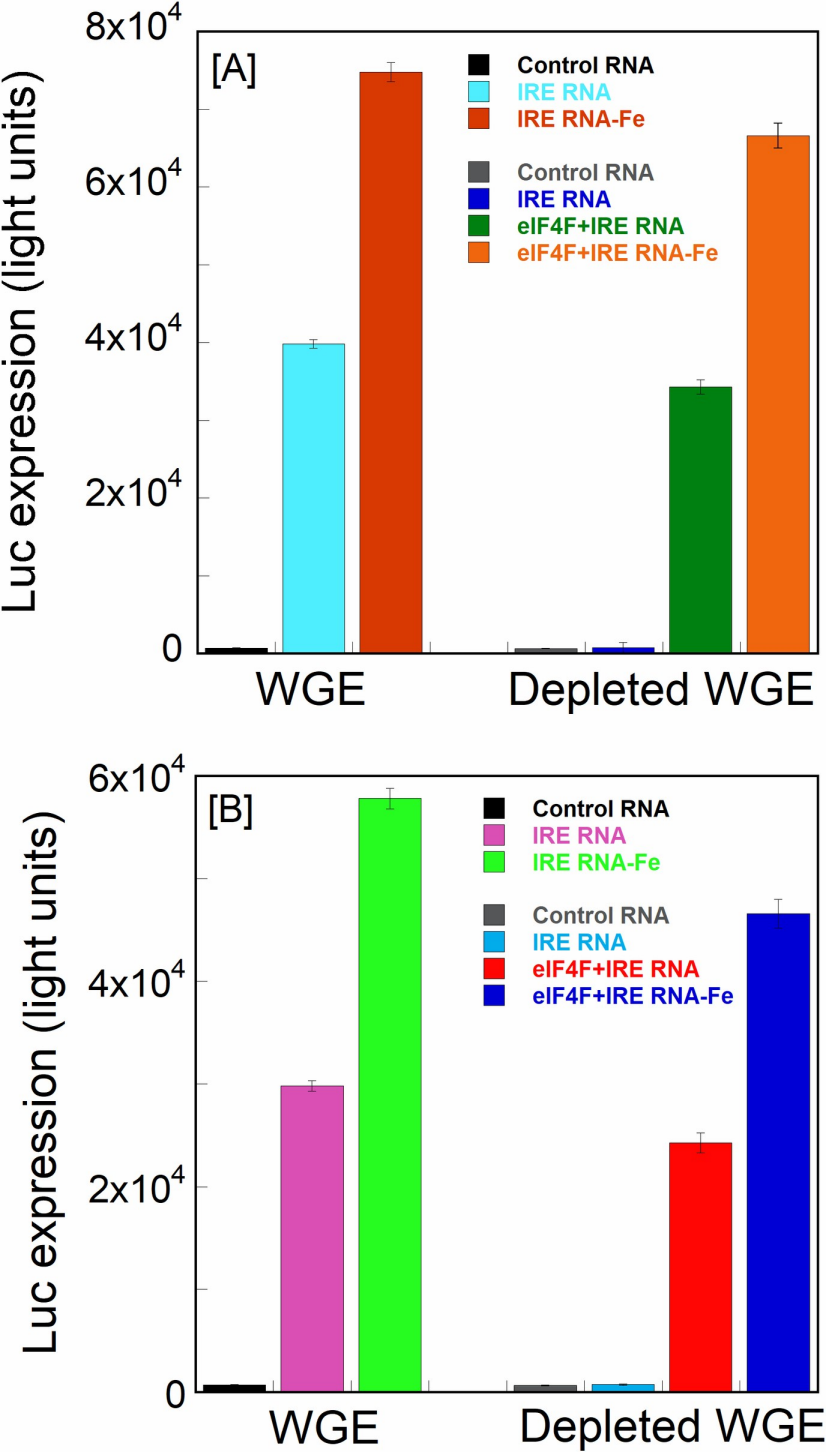
Iron increases protein synthesis of luciferase reporter ferritin and mitochondrial aconitase IRE mRNA constructs in wheat germ extracts. (A) Ferritin IRE luciferase reporter mRNA (capped) translated in wheat germ extracts and eIF4F depleted WG extracts, supplemented with eIF4F (10 nM) and Fe^2+^ (50μM). (B) Aconitase IRE luciferase reporter mRNA (capped) translated in wheat germ extracts and eIF4F depleted WG extracts, supplemented with eIF4F (10 nM) and Fe^2+^ (50μM). Fe^2+^ experiments used anaerobic conditions. Brome mosaic virus RNA (Promega) was used as a control translation assay with WG extract and depleted WG extract. The data are the average of three translation experiments. Error bars indicate standard deviation for three experiments.

To correlate the binding rates of FRT and ACO2 IRE RNA/eIF4F complexes with translation efficiency of the IRE luciferase reporter RNA, eIF4F-depleted cell extract of wheat germ was generated [43]. In order to assess the translation efficiency of the capped IRE RNA, we used complete cell extract of WG and eIF4F-depleted cell extract of WG. As shown in Fig 8, a significant difference was observed between the translation efficiency of FRT and ACO2 IRE RNA in WG extract and depleted WG extract supplemented with eIF4F. Addition of ferritin IRE increased the translation with complete WG extracts. We observed 2-fold increase in translation ferritin IRE luciferase reporter with complete WG extract in the presence of Fe^2+^ ions (Fig 8, Table 2). To initiate protein synthesis, most cellular mRNAs require a presence of m^7^G cap moiety at the 5’-terminus of mRNA for efficient binding with pre-initiation complex and ribosome recruitment [44]. It has been reported that cap directs ferritin RNA synthesis [42]. Ferritin IRE RNA showed high specific binding affinity to eIF4F even without the m^7^G cap [11]; whereas, deleting the cap of IRE mRNA affect the translation [29]. Addition of FRT mRNA to a complete cell extract of WG significantly increased protein synthesis; whereas addition of same RNA to depleted WG extract did not affect protein synthesis under the same conditions. Addition of FRT and ACO2 IRE RNA to the depleted WG extract produced no significant differences in the protein synthesis as compared to the control RNA. Similarly, addition of mitochondrial aconitase IRE RNA to complete WG extract significantly increased translation. We observed 2-fold increase in translation of the aconitase IRE luciferase reporter in the presence of Fe^2+^. In contrast, supplementation of eIF4F in depleted WG extract increased translation for aconitase and ferritin IRE luciferase reporter RNA (Fig 8B, Table 2). We observed 2-fold increase in translation in depleted WG extract supplemented with eIF4F in the presence of Fe^2+^. Supplementation of exogenous eIF4F to the depleted WG extract has resulted in 86% and 82% recovery of translation of capped ferritin and mitochondrial aconitase RNA, confirming the importance of eIF4F in an *in vitro* protein synthesis. Depleted WG extracts data show that IREs do need eIF4F complex for translation and cannot work as IREs element. Furthermore, the observation of 2-fold increase in translation in Fig 8 and Table 2 in the presence of Fe^2+^ is similar to data observed in binding rates in the Fig 3.

**Table 2:** eIF4F supports translation of ferritin and mitochondrial aconitase IRE luciferase reporter.

Ferritin mRNA Mitochondrial Aconitase mRNA				
	LU	Increase (-fold)	LU	Increase (-fold)
**WGE**				
Control RNA	695 ± 45	1	687 ± 47	1
IRE RNA	39813 ± 559	57	29813 ± 519	43
IRE RNA + Fe^2+^	74799 ± 1200	108	57789 ± 996	84
**Dep WGE**				
Control RNA	668 ± 48	1	657 ± 53	1
IRE RNA	765 ± 31	1	733 ± 61	1
eIF4F+IRE RNA	34296 ± 627	51	24298 ± 974	37
eIF4F+IRE RNA + Fe^2+^	66600 ± 1599	100	46708 ± 1389	71

Effect of eIF4F on the protein synthesis of capped IRE mRNA in eIF4F-depleted WG extract. Fold increase is relative to the translation in WGE and Dep WGE with BMV control RNA.

## Discussion

Binding of the eIF4F to IRE RNA is thought to be the rate limiting step in the initiation of protein synthesis [25]. Regulation of the rate of IRE RNA translation is central to iron homeostasis. Elevated concentrations of cellular iron; increase the ratio of the IRE RNA molecules bound to eIF4F protein, and simultaneously, lower the number of IRE RNA molecules bound to IRP [11, 22]. As a result, protein synthesis of the IRE RNA increases. Temperature-dependent kinetic rates for eIF4F interactions with ferritin and mitochondrial aconitase IRE RNA were compared and correlated with protein synthesis. Previous [11] studies showed that ferritin IRE RNA interacts with eIF4F protein, and this interaction plays an important role for successful IRE-dependent translation. Our recent [45] studies on molecular docking and competitive binding analysis showed that IRE RNA specifically binds to eIF4G, while m^7^G cap binds to eIF4E subunit of eIF4F. Using stopped-flow fluorescence, we show the comparative kinetic measurements of FRT and ACO2 IRE binding to eIF4F protein with and without addition of iron. These interactions were measured under pseudo-first order conditions, whereas traces followed a single-exponential kinetic mechanism. The differences in the rapid binding rates accounts largely for the observed selective binding between ferritin and mitochondrial aconitase IRE RNA to the eIF4F protein. Previously [25], we have determined the rates for the reactions of IRP and eIF4F proteins with ferritin IRE RNA. Here, we present the detailed description of the temperature-dependent binding rate of IRE binding to eIF4F protein with and without iron; these interactions have not been reported previously. It was demonstrated that ferritin IRE RNA interacts with eIF4F protein, and iron ions stabilize the complex [11]. Stopped-flow data at different temperatures reveal that binding rates of ferritin IRE RNA to the eIF4F protein is always more than the mitochondrial aconitase IRE RNA binding to eIF4F. Addition of iron to the eIF4F/IRE complex enhanced the binding rate 2-fold and lowered the rate dissociation 3-fold. Significant differences in the association versus dissociation rates are seen in the presence of iron when eIF4F binds to the IRE RNAs. Our results favor the formation and stabilization of eIF4F∙IRE RNA-Fe^2+^ complex, and the rates reveal a mechanism, where eIF4F protein competes kinetically with IRP for IRE RNA binding [11]. Interestingly, iron has more profound effect on the FRT IRE RNA binding to eIF4F, compared to the ACO2 IRE RNA binding. Iron affects both the association and dissociation of FRT and ACO2 IRE binding to eIF4F. This rapid reaction of eIF4F binding to ferritin IRE RNA in the presence of iron suggests that ferritin IRE RNA forms a stable complex more rapidly with the eIF4F protein than to the mitochondrial aconitase IRE RNA. The interactions of the mRNA oligonucleotide hairpin structure induce conformational changes in the structure of the eukaryotic translation initiation factor, leading, subsequently, to an enhanced association between the RNA and the eIF [46, 47]. Iron further stabilizes the eIF4F∙IRE RNA complex via conformational changes produced upon iron binding, similar to those observed for IRP binding to IRE RNA.

The Arrhenius activation energies for the binding of both types of IRE RNAs with eIF4F protein showed a significant difference (*E*_a_ = 52.7 ± 3.4 kJ/mol for FRT and 56.8 ± 2.8 kJ/mol for ACO2 IRE). Addition of iron, lowered the activation energies for eIF4F·FRT IRE and eIF4F·ACO2 IRE complexes. Diminished temperature suggests that not only the IRE RNAs compete in binding to the eIF4F protein in the presence of iron, but also provides a path with a substantially lower energy barrier. Addition of iron to the eIF4F·FRT IRE or eIF4F·ACO2 IRE complexes decreased activation energies, suggesting that iron promotes conformational changes within the eIF4F∙IRE RNA complex. Previously [22, 48], we showed that metal ions facilitate the release of IRP from IRP∙IRE RNA complex, allowing binding of eIF4F, and subsequent increase in the protein synthesis. The lower activation energy for the ferritin IRE RNA suggests that the intermediate achieves a stable conformation more easily as compared to the mitochondrial aconitase IRE RNA intermediate. Binding of IRE RNA to the eIF4F protein induces conformational changes in the protein [45]. These conformational changes promote increased specificity and provide favorable positioning of the stable complex for an effective iron-dependent IRE RNA-driven translation, pivotal in iron regulation. Iron reduces complex dissociation and enhances translation by forming a more stable platform for further assembly of the initiation complex. It has been shown previously [11] that eIF4F and IRP bind to ferritin IRE RNA competitively. Cellular iron level decreases IRE/IRP binding and increases eIF4F·IRE binding affinity, ribosome assembly, and enhances ferritin mRNA translation [11]. Here we further show that addition of exogenous eIF4F increases iron-dependent translation in eIF4F-depleted WG extract. Iron enhances significantly an *in vitro* translation of FRT and ACO2 IRE mRNA in WG extracts and depleted WG extracts supplemented with eIF4F. These results indicate that addition of exogenous eIF4F and iron restores translation in depleted WG extracts, confirming the importance of iron for eIF4F binding to IRE RNA; this interaction favors positively the eIF4F-dependent protein synthesis. Iron-stabilized binding and more rapid formation of the eIF4F∙IRE RNA complex leads to an enhanced *in vitro* protein synthesis of ferritin and mitochondrial aconitase IRE RNA, demonstrating biological importance of this binding during cellular environment changes. These results point to the significance of iron on the eIF4F·IRE RNA complex formation and reflect a kinetic advantage over IRP∙IRE RNA complex.

Structural differences between ferritin and mitochondrial IRE RNAs are reflected in the stability and kinetic differences of the protein/IRE RNA complexes observed here, since eIF4F protein is present in both complexes and only the IRE RNA is differs. IRE RNA structural differences then explain, at least in part, for the significant difference that occur during an *in vitro* iron-induced protein synthesis for IRE RNAs and the differences in their binding [11]. We found that IRE RNA interacts with both proteins IRP and eIF4F in a very rapid reaction. The addition of iron ion lowers affinity and fast dissociation of IRP from IRP/IRE RNA complex. The rapid release of IRP allows for the increased eIF4F association and decreased dissociation rate, resulting in increased protein synthesis of the mRNA. Greater understanding of how the eIF4F and IRP proteins mediate cellular iron distribution may ultimately provide new insights into the processes involved in neurodegenerative diseases, resulting in novel therapies for these diseases.

## References

[1] Costain G, Ghosh MC, Maio N, Carnevale A, Si YC, Rouault TA, et al. Absence of iron-responsive element-binding protein 2 causes a novel neurodegenerative syndrome. Brain Behav. 2019;142(5)(5 1):1195–202. 10.1093/brain/awz072 30915432 PMC6487337

[2] Ward RJ, Zucca FA, Duyn JH, Crichton RR, Zecca L. The role of iron in brain ageing and neurodegenerative disorders. Lancet Neurol. 2014;13(10)(10):1045–60. 10.1016/S1474-4422(14)70117-6 25231526 PMC5672917

[3] Zecca L, Youdim MB, Riederer P, Connor JR, Crichton RR. Iron, brain ageing and neurodegenerative disorders. Nat Rev Neurosci 2004;11(11 5):863–73. 10.1038/nrn1537 15496864

[4] Hare D, Ayton S, Bush A, Lei P. A delicate balance: Iron metabolism and diseases of the brain. Front Aging Neurosci. 2013;5(34):1–19. 10.3389/fnagi.2013.00034 23874300 PMC3715022

[5] Zhou ZD, Tan E-K. Iron regulatory protein (IRP)-iron responsive element (IRE) signaling pathway in human neurodegenerative diseases. Mol Neurodegener. 2017;12(75):1–13. 10.1186/s13024-017-0218-4 29061112 PMC5654065

[6] Pantopoulos K. Iron metabolism and the IRE/IRP regulatory system: an update. Ann N Y Acad Sci. 2004;1012:1–13. 10.1196/annals.1306.001 15105251

[7] Zhang DL, Ghosh MC, Rouault TA. The physiological functions of iron regulatory proteins in iron homeostasis—an update. Front Pharmacol. 2014;5:124. 10.3389/fphar.2014.00124 24982634 PMC4056636

[8] Piccinelli P, Samuelsson T. Evolution of the iron-responsive element. RNA. 2007;13(7):952–66. 10.1261/rna.464807 17513696 PMC1894933

[9] Chen SC, Olsthoorn RCL. Relevance of the iron-responsive element (IRE) pseudotriloop structure for IRP1/2 binding and validation of IRE-like structures using the yeast three-hybrid system. Gene. 2019;710(8 20):399–405. Epub 2019 Jun 12. 10.1016/j.gene.2019.06.012 31200088

[10] Rogers JT, Xia N, Wong A, Bakshi R, Cahill CM. Targeting the Iron-Response Elements of the mRNAs for the Alzheimer’s Amyloid Precursor Protein and Ferritin to Treat Acute Lead and Manganese Neurotoxicity. Int J Mol Sci. 2019;20(4):994. (Feb):1–16. 10.3390/ijms20040994 30823541 PMC6412244

[11] Ma J, Haldar S, Khan MA, Sharma SD, Merrick WC, Theil EC, et al. Fe2+ binds iron responsive element-RNA, selectively changing protein-binding affinities and regulating mRNA repression and activation. Proc Natl Acad Sci U S A. 2012;109(22):8417–22. Epub 2012/05/16. 10.1073/pnas.1120045109 22586079 PMC3365203

[12] Pantopoulos K. Iron regulation of hepcidin through Hfe and Hjv: Common or distinct pathways? Hepatology. 2015;62(6):1922–3. 10.1002/hep.27777 25761647

[13] Kawahara M, Kato-Negishi M, Tanaka K. Amyloids: Regulators of Metal Homeostasis in the Synapse. Molecules. 2020;25(6)(3): 1441. 10.3390/molecules25061441 32210005 PMC7145306

[14] Zhang P, Park HJ, Zhang J, Junn E, Andrews RJ, Velagapudi SP, et al. Translation of the intrinsically disordered protein α-synuclein is inhibited by a small molecule targeting its structured mRNA. Proc Natl Acad Sci U S A. 2020;117(3)(1 21):1457–67. 10.1073/pnas.1905057117 31900363 PMC6983430

[15] Yang X, Williams JK, Yan R, Mouradian MM, Baum J. Increased Dynamics of α-Synuclein Fibrils by β-Synuclein Leads to Reduced Seeding and Cytotoxicity. Sci Rep. 2019;9:17579. 10.1038/s41598-019-54063-8 31772376 PMC6879756

[16] Nakamori M, Junn E, Mochizuki H, Mouradian MM. Nucleic Acid–Based Therapeutics for Parkinson’s Disease. Neurotherapeutics. 2019;16(2)(4):287–98. 10.1007/s13311-019-00714-7 30756362 PMC6554378

[17] Hershey JWB, Merrick WC. The pathway and mechanism of initiation of protein synthesis, in Translational Control of Gene Expression. Sonenberg N, Hershey JWB, Merrick WC, editors: Cold Spring Harbor Laboratory Press, Plainview, NY; 2000. 33–88 p. 10.3727/000000001783992696

[18] Sonenberg N. mRNA 5’ cap binding protein and control of cell growth. eds. Hea JWB, editor: Cold Spring Laboratory Press, Cold Spring Harbor, NY; 1996. 245–69 p.

[19] Gingras AC, Raught B, Sonenberg N. eIF4 initiation factors: effectors of mRNA recruitment to ribosomes and regulators of translation. Annu Rev Biochem. 1999;68:913–63. 10.1146/annurev.biochem.68.1.913 10872469

[20] Tarun SZJ, Sachs AB. Association of the yeast poly(A) tail binding protein with translation initiation factor eIF-4G. EMBO J. 1996;15(24):7168–77. 9003792 PMC452544

[21] Merrick WC. Mechanism and regulation of eukaryotic protein synthesis. Microbiol Rev. 1992;56(2):291–315. 1620067 10.1128/mr.56.2.291-315.1992 PMC372869

[22] Khan MA, Walden WE, Goss DJ, Theil EC. Direct Fe2+ sensing by iron-responsive messenger RNA:repressor complexes weakens binding. J Biol Chem. 2009;284(44):30122–8. Epub 2009/09/02. 10.1074/jbc.M109.041061 19720833 PMC2781567

[23] Walden WE, Selezneva AI, Dupuy J, Volbeda A, Fontecilla-Camps JC, Theil EC, et al. Structure of dual function iron regulatory protein 1 complexed with ferritin IRE-RNA. Science (New York, NY). 2006;314(5807):1903–8. Epub 2006/12/23. 10.1126/science.1133116 17185597

[24] Walden WE, Selezneva A, Volz K. Accomodating variety in iron-responsive elements: Crystal structure of transferrin receptor 1 B IRE bound to iron regulatory protein 1. FEBS Letters. 2012;586:32–5. 10.1016/j.febslet.2011.11.018 22119729

[25] Khan MA, Ma J, Walden WE, Merrick WC, Theil EC, Goss DJ. Rapid kinetics of iron responsive element (IRE) RNA/iron regulatory protein 1 and IRE-RNA/eIF4F complexes respond differently to metal ions. Nucleic Acids Res. 2014;42(10):6567–77. 10.1093/nar/gku248 24728987 PMC4041422

[26] Ke Y, Wu J, Leibold EA, Walden WE, Theil EC. Loops and bulge/loops in iron-responsive element isoforms influence iron regulatory protein binding. Fine-tuning of mRNA regulation? J Biol Chem. 1998;273(37):23637–40. 10.1074/jbc.273.37.23637 9726965

[27] Khan MA. Analysis of ion and pH effects on iron response element (IRE) and mRNA-iroin regulatory protein (IRP1) interactions. *Current Chemical Biology*. 2020;14(2):1–12.

[28] Fletcher L, Corbin SD, Browning KS, Rabel JM. The absence of m7G cap on beta-globin mRNA and alfalfa mosaic virus RNA 4 increases the amounts of initiation factor 3S required for translation. *J Biol Chem*. 1990;265:19582–7. 2246243

[29] Dix DJ, Lin PN, Kimata Y, Theil EC. The iron regulatory region of ferritin mRNA is also a positive control element for iron-independent translation. Biochemistry. 1992;31(10):2818–22. 10.1021/bi00125a024 1547222

[30] Ke Y, Sierzputowska-Gracz H, Gdaniec Z, Theil EC. Internal loop/bulge and hairpin loop of the iron-responsive element of ferritin mRNA contribute to maximal iron regulatory protein 2 binding and translational regulation in the iso-iron-responsive element/iso-iron regulatory protein family. Biochemistry. 2000;39(20):6235–42. Epub 2000/05/23. 10.1021/bi9924765 10821699

[31] Grifo JA, Tahara SM, Morgan MA, Shatkin AJ, Merrick WC. New initiation factor activity required for globin mRNA translation. J Biol Chem. 1983;258(9)(5 10):5804–10. 6853548

[32] Bradford MM. A rapid and sensitive method for the quantitation of microgram quantities of protein utilizing the principle of protein-dye binding. Anal Biochem. 1976;72:248–54. 10.1006/abio.1976.9999 942051

[33] Tibodeau JD, Fox PM, Ropp PA, E.C. T, Thorp HH. The up-regulation of ferritin expression using a small-molecule ligand to the native mRNA. Proc Natl Acad Sci U S A. 2006;103(2):253–7. 10.1073/pnas.0509744102 16381820 PMC1326178

[34] Khan MA, Miyoshi H, Gallie DR, Goss DJ. Potyvirus genome-linked protein, VPg, directly affects wheat germ in vitro translation: interactions with translation initiation factors eIF4F and eIFiso4F. J Biol Chem. 2008;283(3):1340–9. Epub 2007/11/30. 10.1074/jbc.M703356200 18045881

[35] Olsen K, Christensen U, Sierks MR, Svensson B. Reaction mechanisms of Trp120—>Phe and wild-type glucoamylases from Aspergillus niger. Interactions with maltooligodextrins and acarbose. Biochemistry. 1993;32(37):9686–93. 10.1021/bi00088a021 8373772

[36] Slepenkov SV, Darzynkiewicz E, Rhoads RE. Stopped-flow kinetic analysis of eIF4E and phosphorylated eIF4E binding to cap analogs and capped oligoribonucleotides: evidence for a one-step binding mechanism. J Biol Chem. 2006;281(21):14927–38. 10.1074/jbc.M601653200 16540463

[37] Khan MA, Goss DJ. Kinetiuc analysis of phosphorylated and non-phosphorylated eIFiso4E binding to mRNA cap analogues. Int J Biol Macromol. 2018;106:387–95. 10.1016/j.ijbiomac.2017.08.041 28797816

[38] Khan MA, Miyoshi H, Ray S, Natsuaki T, Suehiro N, Goss DJ. Interaction of genome-linked protein (VPg) of turnip mosaic virus with wheat germ translation initiation factors eIFiso4E and eIFiso4F. J Biol Chem. 2006;281(38):28002–10. Epub 2006/08/02. 10.1074/jbc.M605479200 16880203

[39] Khan MAaG, D.J. Translation initiation factor (eIF4B) affects the rates of binding of the mRNA m7G cap analogue to wheat germ eIFiso4F and eIFiso4F-PABP. Biochemistry. 2005; 44:4510–6. 10.1021/bi047298g 15766281

[40] Khan MA, Goss DJ. Poly (A) binding protein enhances the binding affinity of potyvirus VPg to eukaryotic initiation factor eIF4F and activates in vitro translation. Int J Biol Macromol. 2019;121(Jan):947–55. 10.1016/j.ijbiomac.2018.10.135 30342940

[41] Khan MA. Phosphorylation of translation initiation factor eIFiso4E promotes translation through enhanced binding to potyvirus VPg. J Biochem. 2019;165(2)(2 1):167–76. 10.1093/jb/mvy091 30371907

[42] Dix DJ, Lin PN, McKenzie AR, Walden WE, Theil EC. The influence of the base-paired flanking region on structure and function of the ferritin mRNA iron regulatory element. J Mol Biol. 1993;231(2):230–40. Epub 1993/05/20. 10.1006/jmbi.1993.1278 7685392

[43] Gallie D. Cap-Independent translation conferred by the 5’ leader of tobacco etch virus is eukaryotic initiation factor 4G dependent. J Virol. 2001;75:12141–52. 10.1128/JVI.75.24.12141-12152.2001 11711605 PMC116110

[44] Gray NK, Hentze MW. Iron regulatory protein prevents binding of the 43S translation pre-initiation complex to ferritin and eALAS mRNAs. EMBO J. 1994;13(16):3882–91. 8070415 10.1002/j.1460-2075.1994.tb06699.x PMC395301

[45] Khan MA, Malik A, Domashevskiy AV, San A, Khan JA. Interaction of ferritin iron responsive element (IRE) mRNA with translation initiation factor eIF4F. Spectrochimica Acta Part A: Molecular and Biomolecular Spectrscopy. 2020;243(118776):1–10. 10.1016/j.saa.2020.118776 32829157

[46] Wang Y, Sha M, WY R, van Heerden A, Browning K, Goss D. pH-dependent and ligand induced conformational changes of eukaryotic protein synthesis initiation factor eIFiso4F: a circular dichroism study. Biochim Biophys Acta. 1996;1297:207–13. 10.1016/s0167-4838(96)00119-7 8917623

[47] Brazzolotto X, Timmins P, Dupont Y, Moulis JM. Structural changes associated with switching activities of human iron regulatory protein 1. J Biol Chem. 2002;277(14)(4 5):11995–2000. 10.1074/jbc.M110938200 11812787

[48] Khan MA, Walden WE, Theil EC, Goss DJ. Thermodynamic and Kinetic Analyses of Iron Response Element (IRE)-mRNA Binding to Iron Regulatory Protein, IRP1. Sci Rep. 2017 7(1):8532.(8 17):1–11. 10.1038/s41598-017-09093-5 28819260 PMC5561112

